# Jianpi Huazhuo Formula Suppresses Choroidal Neovascularization by Modulating the AKT1/MAPK3/IL6 Axis

**DOI:** 10.1155/joph/5529488

**Published:** 2026-10-23

**Authors:** Liu Yang, Shuqi Zhang, Cui Han

**Affiliations:** ^1^ Senior Department of Ophthalmology Chinese PLA General Hospital Beijing China 301hospital.com.cn

**Keywords:** AKT1, ARMD, CNV, IL6, Jianpi Huazhuo formula, MAPK3

## Abstract

This study elucidates the therapeutic mechanisms of Jianpi Huazhuo formula (JHF) in suppressing laser‐induced choroidal neovascularization (CNV) through the modulation of the AKT1/MAPK3/IL6 signaling pathway. Utilizing a murine CNV model, JHF administration markedly attenuated vascular leakage, as evidenced by fluorescein angiography, optical coherence tomography, and fundus imaging. Integrated network pharmacology pinpointed AKT1, MAPK3, and IL6 as pivotal targets, with functional enrichment analyses highlighting their roles in key processes such as reactive oxygen species response and inflammatory regulation, particularly within the PI3K‐Akt and IL‐17 pathways. Molecular docking validated robust interactions between JHF‐derived compounds (e.g., luteolin and quercetin) and these targets, exhibiting binding energies ranging from −4.0 to −7.41 kcal/mol. Subsequent molecular assays confirmed that JHF significantly reduced AKT1, MAPK3, and IL6 expression at transcriptional and translational levels in RPE‐choroid tissues. Time‐dependent expression profiling further revealed sustained suppression of these targets throughout CNV progression, and in vitro validation in an oxidative stress‐induced ARPE‐19 cell model confirmed the direct regulatory effect of JHF on this signaling axis. These findings demonstrate that JHF exerts its antiangiogenic effects via a multitarget network centered on the AKT1/MAPK3/IL6 axis. The study innovatively delineates JHF’s polypharmacological mode of action, offering novel insights for leveraging traditional Chinese medicine in managing neovascular ocular diseases.

## 1. Introduction

Age‐related macular degeneration (ARMD) represents a predominant cause of irreversible vision loss globally, especially among older adults, with its neovascular subtype—characterized by choroidal neovascularization (CNV)—accounting for the majority of severe visual impairment cases [1]. CNV can be associated with a variety of conditions, including ARMD, pathologic myopia, and inflammatory uveitis. This study focuses on its central role in neovascular ARMD, also known as wet ARMD. CNV entails pathological angiogenesis beneath the retina, resulting in leakage, hemorrhage, and eventual photoreceptor degradation. Current therapeutic standards primarily involve antivascular endothelial growth factor (VEGF) agents, which have transformed patient care by curtailing angiogenesis and minimizing fluid accumulation [2]. Nonetheless, these treatments face substantial limitations, such as the necessity for frequent intravitreal injections, high treatment burdens, heterogeneous patient responses, and the emergence of resistance over time [3]. Furthermore, anti‐VEGF therapies largely focus on a single pathway, potentially neglecting the multifactorial etiology of neovascular ARMD (wet ARMD), which encompasses intricate interactions involving oxidative stress, chronic inflammation, and dysregulated signaling cascades [4, 5]. Consequently, there is a pressing demand for innovative therapeutic strategies that can overcome these drawbacks through multitarget mechanisms, enhanced efficacy, and reduced intervention frequency, ultimately improving long‐term patient outcomes and quality of life.

Within this framework, traditional Chinese medicine (TCM) has gained increasing recognition as a source of complementary and alternative therapies, attributed to its holistic principles and potential for multicomponent, multitarget interventions [6]. Sichong Yang and colleagues provided evidence that Sanhua decoction, a TCM formulation, potentially modulates pathological angiogenesis in ARMD by targeting multiple components within the VEGFA‐associated signaling cascade [7]. TCM formulations, like the Jianpi Huazhuo formula (JHF), typically comprise multiple herbal constituents that may act synergistically to modulate various biological processes, including angiogenesis and inflammation—key drivers of CNV progression [8–10]. Although some preliminary clinical observations and experimental studies have hinted at the potential benefits of TCM in managing retinal disorders [11–13], scientific validation of their mechanisms remains scarce, often due to insufficient integration with modern molecular methodologies. Specifically, the exact targets and pathways through which JHF influences ARMD are poorly delineated, impeding clinical adoption. Thus, closing the divide between traditional empirical knowledge and contemporary evidence‐based science is vital to elucidate the therapeutic promise of JHF and analogous compounds, tackling the overarching challenge of devising innovative, multifaceted treatments for ARMD.

Concentrating on JHF, its utilization in ophthalmic conditions such as ARMD is underpinned by historical practice, yet rigorous scientific inquiry is imperative to decipher its molecular mechanisms. Network pharmacology has surfaced as a potent approach for unraveling the complex actions of TCM by forecasting interactions between drug constituents and disease‐associated targets, thereby furnishing a systematic basis for hypothesis formulation [14]. Prior investigations have leveraged network pharmacology to pinpoint potential JHF targets in diverse settings [15], implying roles in regulating inflammation and oxidative stress; however, these analyses have not been extensively applied to CNV in ARMD. In particular, deficiencies exist in corroborating these predictions via experimental models, like laser‐induced CNV in mice—a well‐validated preclinical model for wet ARMD. Additionally, whereas pivotal signaling pathways such as PI3K‐Akt and IL‐17 are known contributors to angiogenesis [16], their direct modulation by JHF in the CNV context remains unexamined. This underscores the requirement for integrated methodologies that merge computational predictions with in vivo and molecular verifications to affirm the significance of candidate targets, including AKT1, MAPK3, and IL6 [17], which are central to cellular survival, proliferation, and inflammatory responses [18].

Expanding on this, the present study homes in on the hypothesis that JHF curbs CNV by targeting the AKT1/MAPK3/IL6 axis—a pathway implicated in crosstalk with VEGF signaling and involved in vascular permeability and inflammation. Preliminary network pharmacology analyses, as reflected in this work, have identified AKT1, MAPK3, and IL6 as core targets, but their functional contributions to JHF’s mechanism warrant empirical confirmation. The laser‐induced CNV model provides a sturdy foundation for such inquiries, enabling the appraisal of morphological alterations through imaging techniques like optical coherence tomography (OCT) and fluorescein angiography (FFA), alongside molecular changes via quantitative real‐time PCR (qPCR) and Western blotting (WB). Despite progress, the existing literature lacks studies that cohesively connect network pharmacology predictions to molecular docking and experimental validation within a unified structure. Hence, this research aims to remedy these gaps by methodically assessing JHF’s impact on CNV formation and its regulation of the AKT1/MAPK3/IL6 pathway, thereby addressing a critical lacuna in comprehending how multitarget TCM formulations can be leveraged for ARMD therapy.

In summary, this investigation establishes that JHF effectively inhibits laser‐induced CNV in mice, as demonstrated by diminished vascular leakage in FFA imaging, and functions mechanistically through the downregulation of the AKT1/MAPK3/IL6 signaling pathway. By amalgamating network pharmacology, molecular docking, and experimental validation, we verify that JHF’s active components form stable bonds with core targets, resulting in reduced mRNA and protein expression of AKT1, MAPK3, and IL6 in the RPE–choroid complex. These outcomes furnish a thorough explanation for JHF’s multitarget inhibitory effect on CNV, laying a robust groundwork for its potential application in treating wet ARMD.

## 2. Methods

### 2.1. Animals and Ethical Approval

All animal procedures complied with the ARVO Statement for the Use of Animals in Ophthalmic and Vision Research and were approved by the Institutional Animal Care and Use Committee (IACUC) of the affiliated institution. Male C57BL/6J mice (8–10 weeks old, specific pathogen‐free [SPF] grade) were obtained from Vital River Laboratory Animal Technology Co., Ltd. (Beijing, China). Animals were maintained under SPF conditions with a 12‐h light/dark cycle, temperature of 22°C ± 2°C, humidity of 50% ± 10%, and free access to food and water.

### 2.2. CNV Model Induction and Drug Treatment

Mice were randomized into control (no laser, vehicle), laser (CNV model, vehicle), and laser + JHF (CNV model, JHF treatment) groups [19]. CNV was induced under anesthesia (pentobarbital sodium 80 mg/kg), i.p.) using a krypton red laser (647 nm; Lumenis Ltd.) with settings of 50–100 μm spot size, 0.1‐s exposure, and 150–300 mW power [20]. Bubble formation validated model success [21]. The JHF (24. 6 g/kg) was administered via oral gavage once daily from laser induction until Day 14 [22, 23].

### 2.3. In Vivo Pharmacodynamic Assessment

FFA and OCT were conducted with a Micron IV small animal retinal imaging system (Phoenix Research Labs, USA). After the intraperitoneal injection of sodium fluorescein (1%, 2 mL), images were acquired to evaluate vascular leakage [24]. From the FFA images, the CNV leakage areas before and after treatment were measured for both the laser + JHF group and the laser group using Image‐Pro Plus 7.0 software. The relative change rate was calculated according to the following formula: (CNV leakage area before treatment − CNV leakage area after treatment)/CNV leakage area before treatment.

### 2.4. Network Pharmacology Analysis

Active constituents of JHF were identified from TCMSP (oral bioavailability ≥ 30%, drug‐likeness ≥ 0.18), HERB 2.0, and CNKI databases [25]. Drug targets were compiled from HERB, TCMSP, and SwissTargetPrediction; ARMD‐related targets were sourced from GeneCards, MalaCards, and OpenTarget [26]. Overlapping targets underwent Gene Ontology (GO) and Kyoto Encyclopedia of Genes and Genomes (KEGG) enrichment analyses using clusterProfiler (v4.0) [27]. Protein–protein interaction (PPI) networks were generated via STRING and visualized in Cytoscape (v3.9.1) [28].

### 2.5. Molecular Docking

Three‐dimensional structures of core targets (AKT1, MAPK3, IL6) were retrieved from UniProt, and ligand structures (e.g., luteolin, quercetin) were obtained from PubChem. Docking simulations were performed with AutoDock 4.0, and binding energies ≤ −4.0 kcal/mol indicated favorable interactions [29].

### 2.6. Preparation of JHF Freeze‐Dried Powder

The herbal formula JHF was prepared according to the original prescription composition. The mixed crude herbs were extracted by boiling in 10 volumes (v/w) of distilled water for 1 h with constant stirring. The decoction was centrifuged at 3000 × g for 15 min at 4°C, and the supernatant was filtered through Whatman No. 4 filter paper, followed by sequential filtration through 0.45‐μm and 0.22‐μm membrane filters for sterilization. The filtrate was frozen at −80°C for 12 h and then lyophilized at −45 to −55°C for 4–5 days to obtain JHF freeze‐dried powder. The powder was reconstituted in sterile distilled water to a stock concentration of 0.05 g/mL (crude drug equivalent) and stored at −20°C until use.

### 2.7. Cell Culture and Viability Assay

Human retinal pigment epithelial (ARPE‐19; ATCC) cells were cultured in the DMEM/F12 medium supplemented with 10% fetal bovine serum and 1% penicillin–streptomycin at 37°C in a humidified atmosphere of 5% CO_2_. Cells were seeded in 96‐well plates at a density of 5 × 10^3^ cells/well and allowed to attach overnight. To determine the noncytotoxic concentration range, cells were exposed to increasing concentrations of JHF (0, 25, 50, 100, 200 μg/mL crude drug equivalent) for 24 h. Cell viability was assessed using the CCK‐8 assay. Absorbance was measured at 450 nm using a microplate reader. The concentration of JHF corresponding to a 10% inhibitory concentration (IC_10_) was selected for subsequent experiments.

### 2.8. Establishment of an Oxidative Damage Model in ARPE‐19 Cells and Experimental Grouping

ARPE‐19 cells were seeded and subsequently treated with 1250 μg/mL sodium periodate (NaIO_3_) to induce oxidative stress [30]. Based on the determined IC_10_ value of 22.19 μg/mL, JHF was administered at a concentration of 25 μg/mL. Three experimental groups were established: untreated control, NaIO_3_ treatment alone, and cotreatment with NaIO_3_ and JHF. After 24 h of incubation, cells were harvested for the WB analysis of the target proteins.

### 2.9. qPCR

Mice were sacrificed at 0, 1, 3, 7, and 14 days after laser treatment. The eyeballs were immediately enucleated, and the RPE–choroid complex was isolated under a dissecting microscope, snap‐frozen in liquid nitrogen, and stored at −80°C for subsequent analysis. RPE–choroid tissues were homogenized in TRIzol reagent (Invitrogen, USA) for RNA extraction. cDNA was synthesized using a PrimeScript RT kit (Takara, Japan). qPCR was carried out with SYBR Green Master Mix (Applied Biosystems, USA) on a QuantStudio 5 system (Thermo Fisher, USA). The primers of AKT1: forward primer: TCTGCCCTGGACTACTTGC, reverse primer: GTTATCTTGATGTGCCCGTCCT; the primers of MAPK3: forward primer: CTGCCCTCGAAAACCAAGGTG, reverse primer: TCCTCTACTGTGATGCGCTTG; the primers of IL6: forward primer: AGAAAGACAAAGCCAGAGTCC, reverse primer: GCCACTCCTTCTGTGACTCCA. β‐Actin served as the endogenous control, and relative mRNA expression was calculated using the 2^(−ΔΔCt)^ method [31].

### 2.10. WB

Proteins were isolated with RIPA buffer (Beyotime, China) and quantified via BCA assay (Thermo Fisher). Samples were resolved by SDS–PAGE, transferred to PVDF membranes (Millipore, USA) [32] and probed with primary antibodies against AKT1 (Abcam, ab314110), MAPK3 (Abcam, ab32537), IL6 (Abcam, ab233706), and β‐actin (Abcam, ab8227). HRP‐conjugated secondary antibodies and ECL substrate (Millipore) were used for signal detection.

## 3. Results

### 3.1. In Vivo Imaging Evidence of JHF Inhibiting Laser‐Induced CNV

To evaluate the therapeutic effects of the JHF on laser‐induced CNV in mice, imaging analysis of the mouse retina was performed using fundus OCT, fundus photography, and FFA. As shown in Figure 1A, OCT and fundus photography results revealed a normal retinal structure and vascular networks in the control group. In the laser model group, OCT images displayed patchy high‐reflectivity signals beneath the retinal neuroepithelial layer, corresponding to the scar locations observed in fundus photography, indicating successful establishment of the CNV mouse model. FFA images of the laser model group exhibited significant vascular leakage and CNV lesions both before and after treatment. In contrast, FFA images of the laser + JHF treatment group showed markedly reduced fluorescence leakage, suggesting that JHF effectively inhibited abnormal vascular leakage in laser‐induced CNV. According to FFA images, the statistical analysis of the relative change in the CNV leakage area showed that the CNV shrinkage rate in the Laser + JHF group was significantly higher than that in the Laser group (Figure 1B).

**FIGURE 1:**
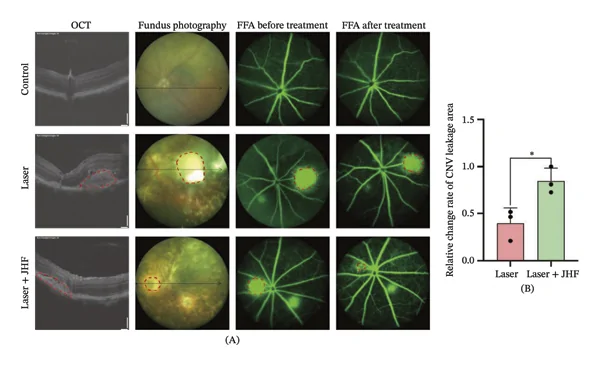
(A) OCT, fundus, and FFA imaging show JHF inhibits laser‐induced CNV in mice (the area within the red‐dotted line represents the CNV). (B) The comparison of the relative change rate of CNV leakage area between the laser group and the laser + JHF group (the relative change rate = (CNV leakage area before treatment‐CNV leakage area after treatment)/CNV leakage area before treatment; *n* = 3, ^∗^ *p* < 0.05).

### 3.2. Network Pharmacology Prediction of Potential Targets and Pathway Enrichment for JHF in Treating ARMD

To elucidate the potential therapeutic targets and mechanisms of JHF for ARMD, we performed a comprehensive network pharmacology study. By integrating drug targets derived from its active components (sourced from the HERB, TCMSP, and SwissTargetPrediction databases; Figure 2A) and known disease targets for ARMD (collected from the GeneCards, MalaCards, and OpenTarget databases; Figure 2B), we established a set of common targets between JHF and ARMD (Figure 2C). GO enrichment analysis of these intersecting targets demonstrated significant enrichment in key biological processes, including response to reactive oxygen species and regulation of the inflammatory response (Figure 2D,E). KEGG pathway analysis further identified significant enrichment in several signaling pathways critical for angiogenesis and inflammation, such as the apoptosis signaling pathway, PI3K‐Akt signaling pathway, and IL‐17 signaling pathway (Figure 2F,G). These findings indicate that JHF likely inhibits CNV through a multitarget mechanism, modulating a network of biological processes and signaling pathways associated with angiogenesis and inflammation.

**FIGURE 2:**
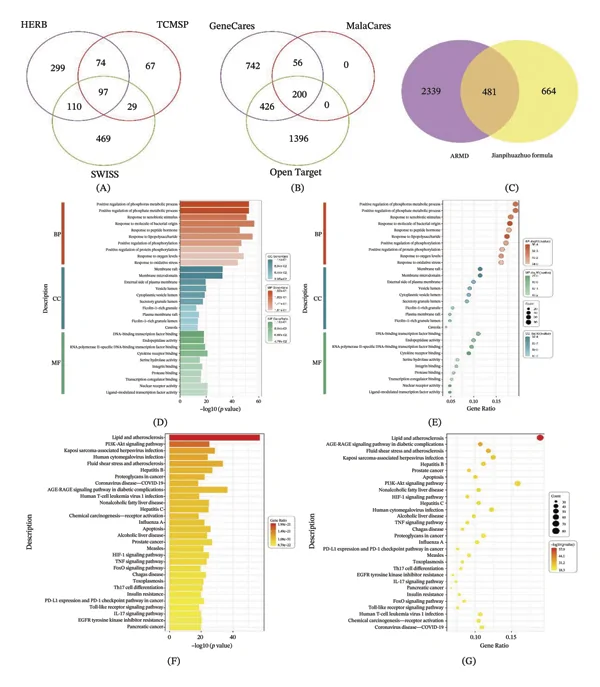
Network pharmacology was used to preliminarily screen the potential targets and mechanisms of the JHF in treating ARMD. (A) Venn diagram of drug targets from three databases. (B) Venn diagram of disease targets from three database sources. (C) The Venn diagram of drug targets vs. disease targets. (D) Intersection target GO enrichment bar chart. (E) GO enrichment bubble diagram of intersection targets. (F) Bar chart of KEGG pathway enrichment analysis of common targets. (G) KEGG pathway enrichment bubble chart of intersection targets.

### 3.3. Core Target Screening and Pathway Association Analysis Unveiling the Network Regulation of JHF

To identify the core targets of JHF in treating ARMD, we further employed three algorithms of the cytoHubba plugin—MCC, degree, and betweenness—to screen for core targets. The intersection of these results was visualized using a Venn diagram (Figure 3A). Subsequently, a PPI network of the core targets was constructed based on the STRING database (Figure 3B). To elucidate the complex relationships among drugs, diseases, active components, core targets, and key signaling pathways, we plotted an integrated network diagram (Figure 3C). Finally, a Sankey diagram illustrating the flow relationships among key components, targets, and pathways was generated for intuitive visualization (Figure 3D). The analysis results indicate that a set of core targets was successfully identified, and their PPI network exhibited dense interactions. Both the network diagram (Figure 3C) and the Sankey diagram (Figure 3D) collectively revealed that JHF may act through multiple active components on several core targets (including AKT1, MAPK3, and IL6), thereby cooperatively regulating signaling pathways closely related to angiogenesis and inflammation, such as the PI3K‐Akt and IL‐17 pathways. This suggests that JHF may exert its inhibitory effect on CNV through a network‐based regulatory mode characterized by “multicomponent, multitarget, and multipathway” interactions.

**FIGURE 3:**
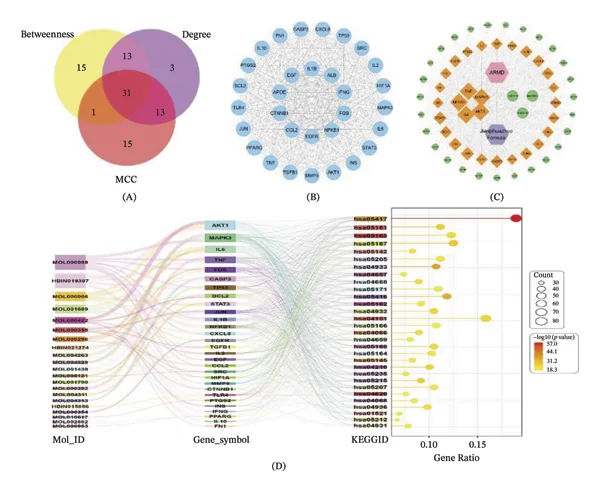
Network analysis identifies core targets of JHF in treating ARMD. (A) Core target Venn diagram. (B) PPI network of key targets. (C) Drug and disease–component–target–pathway network diagram. (D) Component–target–pathway Sankey diagram.

### 3.4. Molecular Docking Validation of Binding Affinity Between Active Compounds of JHF and Core Targets

To computationally validate the predicted interactions between core targets and key active compounds from network pharmacology, we conducted molecular docking simulations using AutoDock 4.0. We assessed the binding of five active constituents (HBIN019307, MOL000006, MOL000098, MOL000422, MOL001689) derived from the JHF to the three identified core targets (AKT1, MAPK3, IL6). The results demonstrate that stable binding conformations were successfully achieved for all five pairs, with calculated binding energies falling within a favorable range of −4.0 to −7.41 kcal/mol. This successful docking confirms, at a computational level, the potential for stable interaction between these compounds and their respective protein targets, thereby supporting the initial network pharmacology analysis and providing a rationale for investigating their modulatory effects on the AKT1/MAPK3/IL6 pathway in subsequent mechanistic studies (see Figure 4).

**FIGURE 4:**
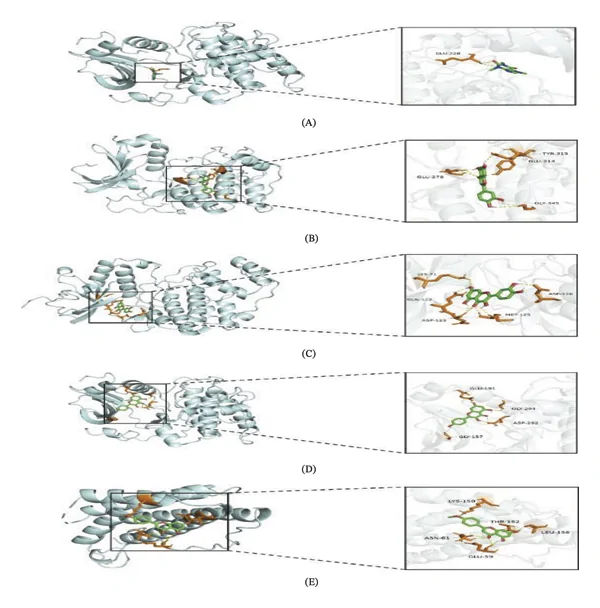
AutoDock 4.0 molecular docking of active compounds with core targets. (A) HBIN019307 (caffeine) docked to AKT1. (B) MOL000006 (luteolin) docked to AKT1. (C) MOL000098 (quercetin) docked to MAPK3. (D) MOL000422 (kaempferol) docked to AKT1. (E) MOL001689 (acacetin) docked to IL6.

### 3.5. Experimental Validation of JHF Downregulating the AKT1/MAPK3/IL6 Pathway in Laser‐Induced CNV

To elucidate the modulatory effect of JHF on the AKT1/MAPK3/IL6 pathway within the context of laser‐induced CNV, we assessed the expression of AKT1, MAPK3, and IL6 via qPCR and WB in murine RPE–choroid complexes (Figure 5A–I). Our analysis revealed a significant upregulation in both mRNA and protein levels of AKT1, MAPK3, and IL6 in the laser group relative to the control group. Crucially, the administration of JHF (laser + JHF group) substantially reversed this upregulation, leading to a marked decrease in the expression of all three targets compared to the laser group. To investigate the temporal expression profiles of AKT1, IL6, and MAPK3, we quantified their mRNA levels at 0, 1, 3, 7, and 14 days postlaser treatment. All three genes were significantly upregulated following laser induction, with expression levels in the laser group remaining consistently above the control baseline throughout the observation period (0–14 days). This pattern exhibited a unimodal trend characterized by an initial increase followed by a subsequent decline. In the laser + JHF group, expression levels were intermediate between those of the laser and control groups, remaining lower than the laser group at all time points, thereby indicating a sustained inhibitory effect of JHF on these three target genes (Figure 5J–L). These results demonstrate that JHF effectively downregulates the expression of AKT1, MAPK3, and IL6 in CNV lesions, implying that its therapeutic suppression of neovascularization may operate through the attenuation of this specific signaling axis.

**FIGURE 5:**
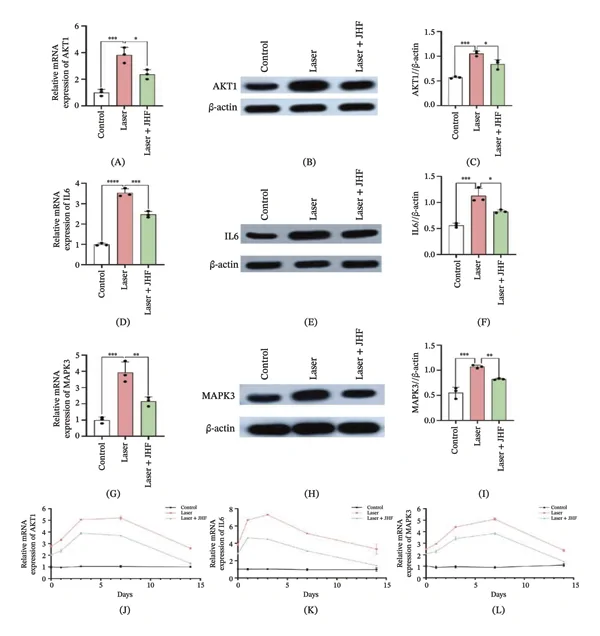
JHF downregulates the AKT1/MAPK3/IL6 pathway in laser‐induced CNV. (A) AKT1 mRNA expression. (B) AKT1 protein bands. (C) AKT1 protein quantification. (D) IL6 mRNA expression. (E) IL6 protein bands. (F) IL6 protein quantification. (G) MAPK3 mRNA expression. (H) MAPK3 protein bands. (I) MAPK3 protein quantification. (J) AKT1 mRNA time‐dependent expression. (K) IL6 mRNA time‐dependent expression. (L) MAPK3 mRNA time‐dependent expression. (*n* = 3, ^∗^ *p* < 0.05, ^∗∗^ *p* < 0.01, ^∗∗∗^ *p* < 0.001, ^*∗∗∗∗*^ *p* < 0.0001).

### 3.6. Experimental Validation of JHF Downregulating the AKT1/MAPK3/IL6 Pathway in ARPE‐19

Figure A presents the CCK‐8 cell viability curve for JHF, revealing an IC50 of 114.2 μg/mL and an IC_10_ of 22.19 μg/mL. Accordingly, a concentration of 25 μg/mL was chosen for subsequent WB analyses. As shown in Figure B–G, NaIO_3_ treatment significantly upregulated the protein expression levels of AKT1, IL6, and MAPK3 relative to the control group. Notably, coadministration of JHF (25 μg/mL) effectively counteracted this effect, resulting in a significant downregulation of all three target proteins compared with the NaIO_3_ model group (see Figure 6).

**FIGURE 6:**
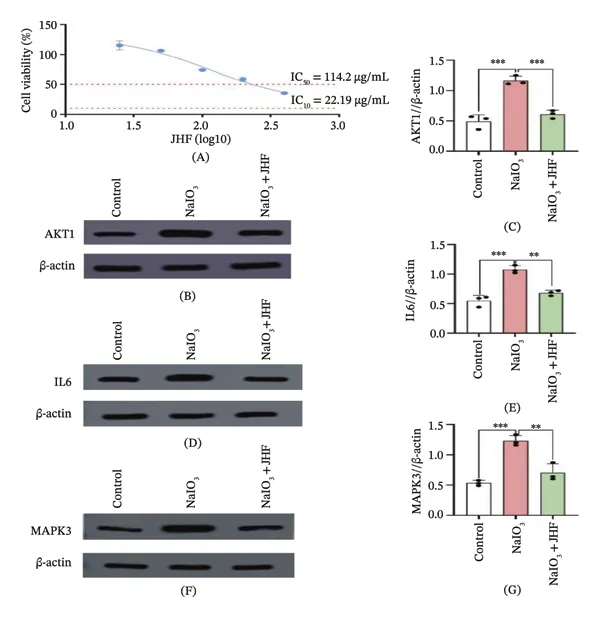
JHF suppresses AKT1, IL6, and MAPK3 expression in ARPE‐19 cells under NaIO_3_‐induced oxidative stress. (A) Cell viability curve of ARPE‐19 cells treated with increasing concentrations of JHF for 24 h. (B, C) Representative Western blot images and quantification of AKT1 protein expression. (D, E) Representative Western blot images and quantification of IL6 protein expression. (F, G) Representative Western blot images and quantification of MAPK3 protein expression. (*n* = 3, ^∗∗^ *p* < 0.01, ^∗∗∗^ *p* < 0.001).

## 4. Discussion

This study comprehensively evaluated the therapeutic effects of JHF on laser‐induced CNV by integrating in vivo and vitro models, network pharmacology, and molecular assays. The key findings indicate that JHF not only markedly suppressed CNV‐related vascular leakage, as evidenced by FFA, but also downregulated the AKT1/MAPK3/IL6 signaling axis, directly addressing the research hypothesis that JHF exerts its actions via a multitarget mechanism. Furthermore, in an NaIO_3_‐induced oxidative damage model in ARPE‐19 cells, JHF (25 μg/mL) significantly downregulated the protein expression of AKT1, MAPK3, and IL6, confirming that the inhibitory effects of JHF on this signaling axis are reproducible in human RPE cells under oxidative stress conditions. This provides direct in vitro evidence supporting the in vivo findings and strengthens the mechanistic link between JHF treatment and the AKT1/MAPK3/IL6 pathway. These outcomes support the potential of JHF as an innovative intervention for ARMD.

The inhibition of CNV by JHF likely arises from its multicomponent modulation of angiogenesis and inflammatory processes. Mechanistic insights from network pharmacology suggest that active compounds in JHF, such as luteolin and quercetin, target key players including AKT1, MAPK3, and IL6, which are implicated in pathways like PI3K‐Akt and IL‐17 [7], thereby regulating oxidative stress and inflammation [33]. Molecular docking simulations reinforced these predictions by demonstrating stable binding between JHF components and the target proteins, with favorable binding energies ranging from −4.0 to −7.41 kcal/mol. This supports the notion that JHF may directly interact with the AKT1/MAPK3/IL6 pathway to attenuate its activity, offering a plausible explanation for the observed efficacy in reducing CNV lesions through a synergistic, multitarget approach. The protective effect on RPE cells under oxidative stress is crucial for AMD therapy [34]. Previous studies have demonstrated that these molecules interact in angiogenesis and inflammation [35, 36]. The present study further reveals that JHF simultaneously targets and downregulates all three key nodes. Temporal expression profiling revealed distinct kinetic patterns for each target. IL6, as an acute pro‐inflammatory cytokine, exhibited a rapid and sharp elevation within the first day postlaser induction (peak ∼6.6‐fold), followed by a swift decline, consistent with its role in initiating the inflammatory cascade. In contrast, AKT1 and MAPK3, which mediate cell survival and proliferative signals, reached their peak expression during the angiogenic active phase (Days 3–7). JHF treatment suppressed all three targets throughout the entire observation period (0–14 days), with the most pronounced inhibition observed during their respective peak windows. This time‐dependent suppression pattern suggests that JHF exerts a multiphase, coordinated intervention—targeting early inflammatory activation (IL6) and later angiogenic signaling (AKT1/MAPK3)—rather than acting uniformly across all time points. Although the classical upstream–downstream signaling relationships among AKT1, MAPK3, and IL6 were not directly validated here, the so‐called “AKT1/MAPK3/IL6 axis” refers to a functional module or network that collectively operates within the PI3K‐Akt, MAPK, and inflammatory signaling pathways and is synergistically downregulated by JHF in CNV.

Our results are consistent with prior literature that highlights the roles of AKT1 and MAPK3 in promoting angiogenesis in CNV models [18] and IL6 in exacerbating ARMD pathology through inflammatory mechanisms [17], aligning with the upregulation of these targets in the laser group. However, some studies present contradictory findings, such as IL6 exhibiting protective effects in certain scenarios, which may be attributed to differences in model systems or experimental timelines. By employing a network pharmacology strategy that aggregates data from multiple databases, our study mitigates biases associated with single‐source predictions. The cellular and molecular signaling mechanisms in ARMD are complex, involving multiple pathways [37]. Nonetheless, it is important to note that much of the existing literature focuses on single‐target interventions, whereas JHF’s multicomponent formulation potentially engages compensatory mechanisms, underscoring the value of herbal medicine in addressing complex diseases like ARMD.

The innovative aspect of this work lies in challenging the paradigm of single‐target therapies for ARMD by proposing a network‐based mechanism for JHF, characterized by multicomponent, multitarget interactions. This provides a theoretical advance in understanding traditional herbal formulas. Moreover, the inclusion of an in vitro oxidative stress model in ARPE‐19 cells bridges the gap between computational predictions and in vivo observations, providing a complete “computational prediction ⟶ in vivo validation ⟶ in vitro mechanistic confirmation” chain of evidence. From a practical standpoint, JHF shows promise as a complementary therapy to enhance the efficacy of anti‐VEGF treatments, with its components like caffeine and kaempferol offering a favorable safety profile that could facilitate clinical translation [38]. The combination of computational and experimental validation also sets a benchmark for future research on complex mixtures. This study has limitations: (1) The laser‐induced CNV model, while effective, may not fully recapitulate the chronic aspects of human ARMD. (2) While our study focused on wet ARMD, targets derived from public databases may encompass certain targets associated with dry AMD. This represents a typical limitation in bioinformatics‐based research, yet it does not undermine the core conclusions pertaining to CNV. (3) While the ARPE‐19 cell experiments confirmed that JHF directly modulates AKT1/MAPK3/IL6 expression in human RPE cells, we acknowledge that the specific in vivo cell types responsible for the observed protein changes in the RPE‐choroid complex remain unidentified. Due to experimental limitations, we were unable to perform cell colocalization experiments such as multiplex immunofluorescence and therefore could not definitively determine which specific cell types were responsible for the observed protein changes. In future in‐depth studies, we plan to utilize single‐cell resolution techniques available on advanced experimental platforms (e.g., single‐cell RNA sequencing or multiplex immunohistochemistry) to precisely map protein changes to specific cell types. (4) FFA, as a two‐dimensional imaging modality, quantifies the area of fluorescein leakage rather than the absolute volume of CNV. Consequently, the present measurements reflect vascular permeability and the severity of leakage rather than the actual neovascular mass. Future studies utilizing OCT angiography (OCTA) or choroidal flat mounts stained with isolectin B4 or CD31 will be required to precisely quantify neovascular volume and vascular coverage, thereby providing a more comprehensive evaluation of the antiangiogenic efficacy of JHF. In addition, long‐term toxicity studies to evaluate the safety of JHF, as well as the use of gene knockout models to clarify the specific role of the AKT1/MAPK3/IL6 pathway and thereby optimize the formulation, will be implemented in future research. Vascular endothelial cells play a pivotal role in neovascularization. Accordingly, further investigations employing human choroidal endothelial cells are warranted to confirm the antiangiogenic activity of JHF and to determine whether the AKT1/MAPK3/IL6 axis is also implicated in mediating endothelial cell responses.

## 5. Conclusion

In conclusion, this study establishes that JHF inhibits CNV through the modulation of the AKT1/MAPK3/IL6 pathway, presenting a multitarget therapeutic strategy for ARMD.

## Author Contributions

Cui Han designed the study, Liu Yang performed data collection and management; Liu Yang and Shuqi Zhang performed data analysis and interpretation; and Liu Yang wrote, and Shuqi Zhang reviewed the manuscript.

## Funding

There was no funding.

## Conflicts of Interest

The authors declare conflicts of interest.

## Data Availability

The data that support the findings of this study are available on request from the corresponding author. The data are not publicly available due to privacy or ethical restrictions.
